# Multifaceted therapeutic potential of saponins in type 2 diabetes mellitus: mechanisms targeting insulin resistance and diabetic complications

**DOI:** 10.3389/fphar.2026.1793697

**Published:** 2026-05-07

**Authors:** Yuan Yuan, Ke-Ying Shou, Si-Qi Wu, Jing-Yu Zhao, Yao Liu, Ya-Jun Wang, Hua-Song Luo, Wen-Ting Dai

**Affiliations:** 1 College of Pharmaceutical Sciences, Zhejiang University of Technology, Hangzhou, Zhejiang, China; 2 Zhejiang Key Laboratory of Green Manufacturing Technology for Chemical Drugs, Deqing, Zhejiang, China; 3 Key Laboratory for Green Pharmaceutical Technology and Equipment (Zhejiang University of Technology) of Ministry of Education, Deqing, Zhejiang, China; 4 State Key Laboratory of Green Chemical Synthesis and Conversion, Hangzhou, Zhejiang, China; 5 Department of Traditional Chinese Medicine Massage, Hangzhou Hospital of Traditional Chinese Medicine, Hangzhou, Zhejiang, China

**Keywords:** diabetic complications, insulin resistance, organ protection, saponin, type 2 diabetes mellitus

## Abstract

**Background:**

Type 2 diabetes mellitus (T2DM) is a complex metabolic disorder that poses management challenges due to the limited efficacy and adverse effects of current therapies. Saponins, a class of bioactive phytochemicals widely distributed in medicinal plants and other natural sources, exhibit antidiabetic activities through multi-target mechanisms, have emerged as promising therapeutic candidates for T2DM. This review systematically summarizes the pharmacological mechanisms and therapeutic potential of saponins in management of T2DM and its complications.

**Materials and methods:**

Studies involving molecular, cellular, animal, and clinical studies of saponins in T2DM and its complications were screened and analyzed to provide a systematic overview. All the literature information in this review was collected from relevant literature published up to 2025 from the scientific databases, including PubMed, Web of Science, and China National Knowledge Infrastructure (CNKI).

**Results:**

The pathogenesis of T2DM is characterized by insulin resistance, β-cell dysfunction, genetic susceptibility, gut microbiota dysbiosis, and metabolic disturbances, leading to hyperglycemia and systemic complications. This review demonstrates that saponins exert multifaceted antidiabetic effects through modulating key signaling pathways, including Keap1/Nrf2, AMPK/PI3K/Akt, and NF-κB. Furthermore, saponins demonstrate significant organ-specific protection against major diabetic complications, such as nephropathy, cardiomyopathy, neuropathy, and retinopathy.

**Conclusion:**

Saponins represent a potent class of bioactive metabolites with multi-target therapeutic potential for T2DM and its complications. Their ability to simultaneously modulate metabolic, inflammatory, oxidative, mitochondrial, and gut microbiota-related pathways highlights clear advantages over single-target therapies. A growing body of research has underscored its potential clinical significance in T2DM, providing essential evidence for developing effective and safe therapeutic strategies.

## 1 Introduction

T2DM has emerged as a global health crisis, characterized by insulin resistance (IR) and progressive β-cell dysfunction (Taylor, 2013). The International Diabetes Federation reports that 589 million adults are living with diabetes, the majority of whom have T2DM (Schwarz, 2025). This epidemic is promoted by modern lifestyle factors, including obesity, physical inactivity, and aging populations (Sun et al., 2022). Furthermore, chronic hyperglycemia creates a self-perpetuating cycle of metabolic deterioration through glucotoxic effects on pancreatic β-cells and insulin-sensitive tissues (Liu et al., 2022). Without effective intervention, patients inevitably develop devastating complications such as cardiovascular disease, nephropathy, and retinopathy, which account for the majority of diabetes-related morbidity and mortality (Davies et al., 2018). These concerning findings emphasize the critical need for novel therapeutic approaches capable of disrupting this pathological cycle. While lifestyle modification remains the cornerstone of T2DM management, poor patient adherence severely limits its long-term pharmacological effects (Zheng et al., 2017). Current pharmacotherapies, though widely used, have significant drawbacks. Metformin, the first-line medication, frequently causes gastrointestinal distress (Foretz et al., 2014), while insulin therapy carries risks of hypoglycemia and weight gain (Cahn et al., 2015). Moreover, many existing drugs target single pathways and fail to address the multifactorial nature of T2DM, which involves complex interactions between metabolic dysregulation, chronic inflammation, and oxidative stress. These limitations highlight the need for novel therapeutic agents that can simultaneously modulate multiple pathological processes with fewer side effects.

Natural products, particularly saponins, have recently gained attention as promising candidates for T2DM treatment. These bioactive metabolites are abundantly present in medicinal botanical plants like *Panax ginseng* C. A. Mey. (Araliaceae), *Astragalus membranaceus* (Fisch.) Bunge (Fabaceae), *Panax notoginseng* (Burkill) F.H. Chen (Araliaceae), and *Polygonatum sibiricum* Redouté (Asparagaceae), as well as in certain animal and marine organisms (e.g., sea cucumbers and starfish) (Osbourn et al., 2011). Structurally, saponins are composed of hydrophilic sugar moieties (glycones) linked via glycosidic bonds to hydrophobic aglycones (sapogenins). Based on the chemical structure of the aglycone, saponins are categorized into two major classes: triterpenoid saponins (featuring a 30-carbon triterpene skeleton conjugated with hydrophilic glycosides) and steroidal saponins (characterized by a 28-carbon spirostane or isospirostane aglycone bound to sugar groups) (Moses et al., 2014). Their amphiphilic nature allows interaction with cellular membranes and modulation of various receptors, resulting in pleiotropic effects including improved insulin sensitivity, enhanced β-cell function, and reduced inflammation. For example, ginsenoside Rb1 (Rb1) ameliorates IR through peroxisome proliferator-activated receptor γ (PPARγ) activation (Ding et al., 2023), while astragaloside IV (AS-IV) protects against diabetic nephropathy by inhibiting the TGF-β/Smad pathway (Shen et al., 2023a). Such multi-target actions position saponins as ideal candidates for addressing the complex pathophysiology of T2DM.

While numerous reviews have documented the anti-diabetic properties of saponins, they have largely focused on individual compound classes or isolated mechanisms, remaining descriptive and lacking critical analysis of the full spectrum of diabetic complications or pharmacological evidence quality. Despite promising findings, critical gaps persist, including fragmented research on individual metabolites, absent systematic evaluation of structure-activity relationships across saponin subclasses, scarce translational research addressing pharmacokinetic challenges, and unexplored synergistic effects within the interconnected networks underlying T2DM pathogenesis. The present review advances beyond prior work by offering a comprehensive and critical synthesis encompassing both triterpenoid and steroidal saponins from diverse botanical sources. It integrates the latest mechanistic insights including mitochondrial dynamics, ferroptosis, pyroptosis, and gut-organ axis regulation, systematically examining therapeutic effects on core metabolic defects and all major diabetic complications. By critically evaluating evidences across molecular, cellular, animal, and clinical studies and identifying key research directions, this work provides a scientific foundation for translating these metabolites into effective, safe, and affordable therapeutic strategies for T2DM.

## 2 Literature search

To ensure comprehensiveness, transparency, and reproducibility, a systematic literature search was conducted following established guidelines. Multiple electronic databases were searched to capture both English and Chinese language publications, including PubMed, Web of Science, and the China National Knowledge Infrastructure (CNKI). The search was limited to literature published from January 2010 to December 2025, focusing on recent advances while ensuring sufficient coverage of foundational studies in the field.

A combination of Medical Subject Headings (MeSH) terms and free-text keywords was employed. The core search strategy for PubMed was as follows, and was adapted accordingly for other databases: (“Saponins” [MeSH] OR “Ginsenosides” [MeSH] OR “saponin” [Title/Abstract] OR “saponins” [Title/Abstract] OR “ginsenoside” [Title/Abstract] OR “ginsenosides” [Title/Abstract] OR “astragaloside” [Title/Abstract] OR “platycodin” [Title/Abstract] OR “dioscin” [Title/Abstract] OR “notoginsenoside” [Title/Abstract] OR “compound K” [Title/Abstract]) AND (“Diabetes Mellitus, Type 2” [MeSH] OR “Diabetes Complications” [MeSH] OR “insulin resistance” [MeSH] OR “type 2 diabetes” [Title/Abstract] OR “T2DM” [Title/Abstract] OR “insulin resistance” [Title/Abstract] OR “diabetic nephropathy” [Title/Abstract] OR “diabetic kidney disease” [Title/Abstract] OR “diabetic cardiomyopathy” [Title/Abstract] OR “diabetic neuropathies” [MeSH] OR “diabetic neuropathy” [Title/Abstract] OR “diabetic retinopathy” [MeSH] OR “diabetic retinopathy” [Title/Abstract] OR “non-alcoholic fatty liver disease” [Title/Abstract] OR “NAFLD” [Title/Abstract]). For Chinese databases, equivalent search terms in Chinese were used, including: (“皂苷” OR “人参皂苷” OR “黄芪甲苷” OR “桔梗皂苷” OR “薯蓣皂苷”) AND (“2型糖尿病” OR “胰岛素抵抗” OR “糖尿病并发症” OR “糖尿病肾病” OR “糖尿病心肌病” OR “糖尿病神经病变” OR “糖尿病视网膜病变”).

**FIGURE 1:**
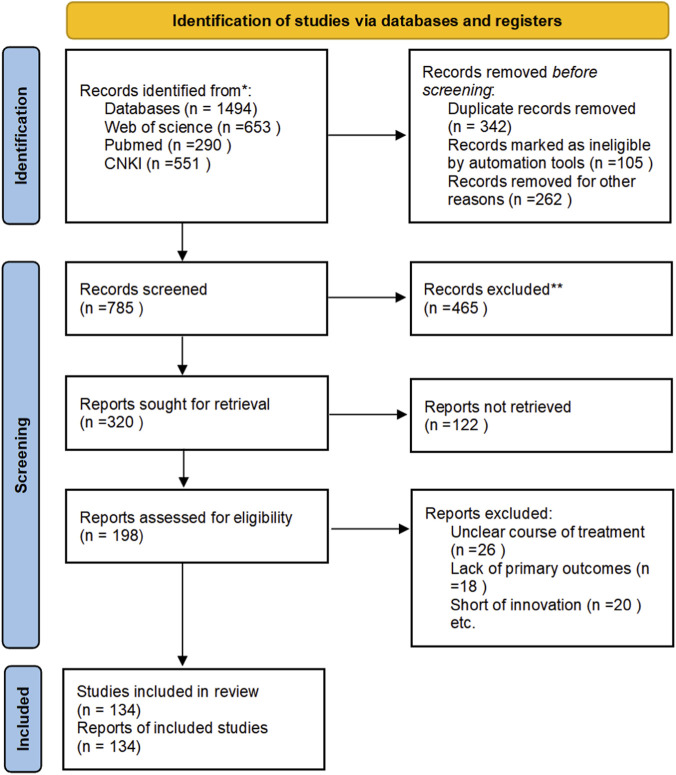
The preferred reporting items for systematic reviews and meta-analyses flow diagram for study selection.

From the included studies, key information was extracted, including saponin type, botanical source with taxonomic verification, study model, dosing regimen, pharmacological effects, and molecular mechanisms. Where available, details regarding plant part used, extraction method, and standardization markers were documented. A considerable proportion of studies lacked comprehensive reporting of these parameters, which represents a limitation affecting the reproducibility and comparative analysis of the evidence - a challenge widely recognized in natural product research. The initial search identified records across all databases; after duplicate removal, title and abstract screening, and full-text eligibility assessment, the final selection of studies was presented in a PRISMA flow diagram (Figure 1).

## 3 Overview of bioactive saponins

**FIGURE 2:**
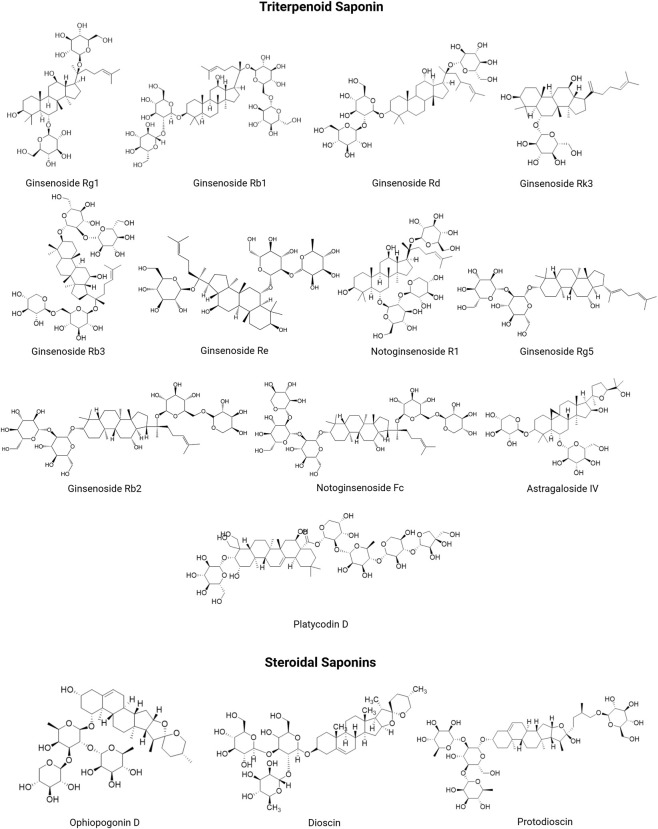
Structure and classifications of saponin metabolites. (Created using ChemDraw).

Saponins represent a structurally diverse class of secondary metabolites characterized by glycosidic linkages between triterpenoid or steroidal aglycones and sugar moieties, broadly classified into triterpenoid (predominantly in dicotyledons like Fabaceae and Araliaceae) and steroidal types (mainly in monocotyledons such as Solanaceae and Liliaceae) (Osbourn et al., 2011). Their distribution across plant tissues (roots, stems, leaves, etc.) exhibits pronounced species specificity, exemplified by AS-IV in Astragalus (immunomodulatory/antitumor) (Yang et al., 2013; Zhou C. et al., 2023), glycyrrhizic acid in *Glycyrrhiza glabra* L. [Fabaceae] (antiviral) (Chrzanowski et al., 2020), and over 170 ginsenosides in *Panax ginseng* (anti-inflammatory/antitumor) (Alolga et al., 2020; Xiaodan and Ying, 2022) (see Figure 2; Table 1 for structural details, classifications, and sources of saponins). For instance, ginsenoside Rb1, a prototypical dammarane-type saponin, exhibits an oral bioavailability below 1% in rodents, yet still produces dose-dependent pharmacological effects *in vivo*. This paradox is explained by its prodrug nature: Rb1 is poorly absorbed in its intact form but undergoes sequential deglycosylation by intestinal bacteria to generate more bioavailable metabolites. Specifically, Rb1 is metabolized via the ginsenoside Rd pathway, undergoing stepwise deglycosylation to Rd, then ginsenoside F2, and ultimately to compound K, the principal absorbable and pharmacologically active metabolite (Xu et al., 2017; Shen et al., 2013). Following intravenous administration in rodents, ginsenoside Rd exhibits rapid and extensive tissue distribution, with the highest concentrations in the lung, followed by the liver, kidney, and intestine, while brain exposure remains low, likely due to the blood-brain barrier. The compound is largely cleared within 24 h, primarily via urinary excretion, and its plasma concentration-time profile fits a two-compartment open model with linear pharmacokinetics (Sun et al., 2012). This necessity represents both a challenge and an opportunity, suggesting the potential for combining saponins with prebiotics or designing prodrugs to enhance their pharmacological effects.

**TABLE 1:** Origin and concentration of saponins in different plants.

Classification	Plant species	Source materials	Saponin	Molecular formula	Extraction method	Yield (mg/g DW)^1^	Ref
Triterpenoid saponins	*Panax ginseng* C. A. Mey. (Araliaceae)	Ginseng root	Ginsenoside Rg1	C_42_H_72_O_14_	HPLC	4.50 ± 0.10	Lin et al. (2013)
			Ginsenoside Rb1	C_54_H_92_O_23_	HPLC	4.50 ± 0.12	Lin et al. (2013)
			Ginsenoside Rb2	C_53_H_90_O_22_	HPLC	1.63 ± 0.03	Lin et al. (2013)
			Ginsenoside Re	C_48_H_82_O_18_	HPLC	3.04 ± 0.04	Lin et al. (2013)
			Ginsenoside Rb3	C_53_H_90_O_22_	HPLC	2.30 ± 0.22	Lin et al. (2013)
			Ginsenoside Rd	C_48_H_82_O_18_	HPLC	0.70 ± 0.12	Lin et al. (2013)
		*P. notoginseng* root	Notoginsenosides R1	C_47_H_80_O_18_	Enzyme-assisted technology	15.99 ± 0.48	Li N. et al. (2022)
	*A. membranaceus* (Fisch.) Bge. (AM),	AM root	Astragaloside IV	C_41_H_68_O_14_	UPLC	0.456 ± 0.009	Li et al. (2019)
			Astragaloside II	C_43_H_70_O_15_	UPLC	0.324 ± 0.007	Li et al. (2019)
	*Platycodon grandiflorum* (Jacq.) A. DC. (Campanulaceae)	Dried roots of PG	Platycodin D	C_57_H_92_O_28_	Mechanochemical-assisted extraction	7.16 ± 0.14	Wu and Xi (2020)
Steroidal saponins	*Dioscorea opposita* Thunb. (Dioscoreaceae)	*D. rotundata* tubers	Dioscin	C_45_H_72_O_16_	HPTLC	11.7 ± 9.4	Lebot et al. (2019)
	*Ophiopogon japonicus* (Thunb. ex L. f.) Ker Gawl. (Asparagaceae)	Fibrous roots of Hang maidong (HMD) and Chuan maidong (CMD)	Ophiopogonin D	C_44_H_70_O_16_	HPLC-ELSD	0.209 ± 0.005	Li et al. (2016)

1 Yield of the metabolite quantified in dried plant material (mg/g dry weight).

## 4 Pathogenesis of T2DM and its complications: key molecular targets

### 4.1 Core pathogenic mechanisms of T2DM

T2DM is a multifactorial metabolic disorder characterized by IR in peripheral tissues (skeletal muscle, liver, and adipose) and progressive β-cell dysfunction, leading to impaired insulin secretion. IR results from defects in insulin signaling transduction, dysregulation of glucose transporters (e.g., GLUT4), and lipotoxicity-induced metabolic disturbances. Compensatory hyperinsulinemia initially maintains glucose homeostasis, but prolonged β-cell hypersecretion leads to exhaustion, with 25%–40% of insulin-resistant individuals progressing to hyperglycemia (Weir and Bonner-Weir, 2013). Visceral obesity exacerbates IR through chronic low-grade inflammation and disruption of insulin signaling pathways, establishing a key link between adiposity and metabolic dysfunction.

### 4.2 Genetic determinants of T2DM susceptibility

Genome-wide association studies (GWAS) have identified over 400 risk loci implicating pathways in insulin secretion, glucose/lipid metabolism, and inflammatory signaling (Mahajan et al., 2022). Notable examples include transcription factor 7-like 2 (TCF7L2) variants, which impair glucagon-like peptide-1 (GLP-1)-mediated insulin secretion by reducing β-cell glucose sensitivity, and PPARγ loss-of-function mutations, which disrupt adipocyte differentiation, promote ectopic lipid deposition, and worsen systemic IR (Wu et al., 2021). These genetic insights underscore the polygenic nature of T2DM and its intersection with metabolic and inflammatory pathways.

### 4.3 Gut microbiota dysbiosis in T2DM pathogenesis

High-fat diets and metabolic dysfunction alter gut microbial composition, contributing to intestinal barrier impairment and systemic inflammation. Metagenomic analyses reveal consistent microbiota shifts in T2DM patients, including an increased Firmicutes/Bacteroidetes ratio and depletion of butyrate-producing bacteria (e.g., *Faecalibacterium prausnitzii*) (Thingholm et al., 2019; Forslund et al., 2015), which can be an effective pharmacological approach for T2DM by inducing nucleosome repositioning within nuclear-encoded mitochondrial genes (Henagan et al., 2015). Fecal microbiota transplantation from lean donors improves insulin sensitivity in metabolic syndrome patients, highlighting the microbiota’s role in energy metabolism and IR (Vrieze et al., 2012). Targeted modulation of microbial communities and their metabolites (e.g., short-chain fatty acids) represents a promising therapeutic avenue.

### 4.4 Systemic complications and multi-organ dysfunction in T2DM

T2DM induces both acute (e.g., ketoacidosis) and chronic complications, the latter driven by hyperglycemia-induced oxidative stress and advanced glycation end (AGE)-product accumulation. Chronic complications include microvascular damage (retinopathy, nephropathy, neuropathy), macrovascular disease (accelerated atherosclerosis), and neurodegenerative risks (e.g., Alzheimer’s disease via IR and neuroinflammation) (Giacco et al., 2010; Luo et al., 2022). The global economic burden of T2DM exceeds 1 trillion dollars annually, requiring safer, multi-target therapies. Natural saponins, with their dual modulation of IR (via AMPK/PI3K/Akt) and inflammation (via NF-κB suppression), offer a promising strategy to address these unmet clinical needs.

## 5 Pharmacological effects and mechanisms of bioactive saponins in T2DM

### 5.1 Regulation of glucose metabolism: hepatic gluconeogenesis inhibition

**FIGURE 3:**
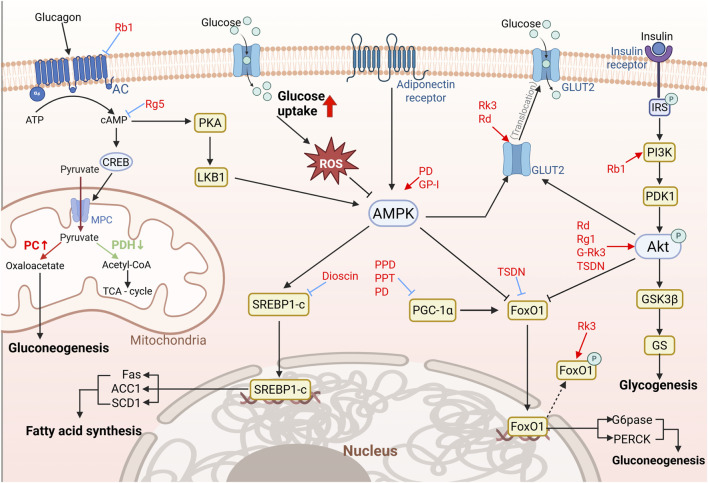
Molecular mechanisms of saponin-mediated inhibition of hepatic gluconeogenesis (Created using BioRender). Bioactive saponins exert multiple targeted suppressions of hepatic gluconeogenesis through coordinated modulation of key metabolic pathways. First, saponins disrupt the cAMP/CREB axis by inhibiting AC activity and CREB phosphorylation, thereby reducing MPC1 expression and limiting pyruvate influx into mitochondria. Second, saponins directly suppress the activity of PC and PDH, reducing the supply of gluconeogenic precursors. Third, saponins (e.g., dioscin) activate AMPK, which concurrently inhibits lipogenesis via SREBP1c downregulation and enhances insulin sensitivity through PI3K-Akt/GSK3β signaling, promoting glycogen synthesis. Finally, saponins fine-tune mitochondrial function and redox balance by regulating PGC-1α and FoxO1, further attenuating glucose production.

Hepatic glycogen synthesis is a central regulator of glucose homeostasis, preventing postprandial hyperglycemia through glucose storage and maintaining fasting euglycemia via glycogenolysis (Petersen et al., 2017). This process is precisely regulated by nutritional status, hormonal signals (e.g., insulin and glucagon), and energy demands (Hatting et al., 2017). In T2DM, IR disrupts insulin signaling, leading to uncontrolled gluconeogenesis and fasting hyperglycemia. Clinical studies indicate that hepatic gluconeogenic flux in T2DM exceeds healthy controls by 40%–200%, underscoring its pathogenic centrality (Shah and Wondisford, 2020). Herein, ginsenosides Rb1 and Rg5 ameliorate T2DM by targeting hepatic gluconeogenesis through multiple mechanisms (Figure 3; Table 2). Rb1 inhibits adenylate cyclase (AC), while Rg5 inhibits succinate accumulation in hepatocytes by combating fatty acid oxidation, thereby reducing cAMP accumulation by blocking succinate/HIF-1α induction (Xiao et al., 2017; Lou et al., 2019). This cascade downregulates mitochondrial pyruvate carrier 1 (MPC1), CREB-driven pyruvate carboxylase (PC) transcription, and restores pyruvate dehydrogenase (PDH) activity, thereby blocking the conversion of pyruvate to oxaloacetate and the supply of gluconeogenic substrates. Research further reveals that p52, a key activator of the nuclear factor kappa-B (NF-κB) signaling pathway, is abnormally activated in the livers of T2DM models, leading to the downregulation of phosphodiesterase 4B (PDE4B) expression, which potentiates glucagon-driven hyperglycemia. Rb1 inhibits p52 nuclear translocation and Rg5 stabilizes PDE4, collectively preventing hepatic glucose output (Xiao et al., 2017; Zhang et al., 2019). These findings elucidate a saponin-mediated gluconeogenesis suppression, highlighting their potential as targeted therapeutics for T2DM.

**TABLE 2:** Mechanism of action of saponin monomers in the treatment of T2DM.

Plant species	Saponin	Study model	Dosing	Pharmaceutical effects	Mechanism	Ref
*Panax ginseng* C. A. Mey. (Araliaceae)	Ginsenoside Rg1	HFD T2DM C57BL/6 mice	Diet, 50 mg/kg for 8 weeks	Inhibit glucagon-induced hepatic gluconeogenesis	Reduce hepatic glucose output: p-Akt↑, PEPCK↓, G6pase↓	Liu et al. (2017)
		Insulin resistance model of HepG2 cells	20, 40, 80 μM for 12 h	Improve insulin resistance	① Increased glucose uptake: ROX3↓, ROS↓, p38 MAPK↓ ② Reduce glucose output: p-Akt↑, GSK3β↓	Fan et al. (2019)
	Ginsenoside Rb1	HFD T2DM Kkay mice	i.g. 200 mg/kg for 4 weeks	Regulate the composition and function of the intestinal flora	*Firmicutes/Bacteroidetes* ratio↓, the abundance of beneficial bacteria↑, and the abundance of bacteria related to metabolic disorders↓	Zhou R. et al. (2023)
		Adipose-derived stem cells and primary macrophages	100 μM for 12 h	① Improve insulin resistance ② Suppress the inflammatory response	① IRS1↑, PI3K↑ ② PPARγ↑, MCP-1↓, TNF-α↓, IL-1β↓, IL-10↑	Ding et al. (2023)
		Male C57BL/6J mice	i.g. 20, 50 mg/kg	Inhibit hepatic gluconeogenesis	AC↓, cAMP↓, CREB inactivation, MPC1↓	Lou et al. (2019)
	Ginsenoside Rb2	HFD obesity C57BL/6J mice	i.p. 40 mg/kg for 10 day	Improve insulin resistance	① IRS-1/PI3K/AKT signaling pathway activation ① MAPK↓, JNK↓, NF-κB↓	Dai et al. (2018)
	Ginsenoside Rk3	HFD/STZ T2DM C57BL/6 mice	i.g. 10, 30, 60 mg/kg for 4 weeks	Inhibit hepatic gluconeogenesis	① AMPK↑, ACC↓, FAS↓, SREBP-1↓ ②p-Akt↑, p-FoxO1↑, GLUT2↑, PEPCK, G6pase↓	Liu et al. (2019)
	Ginsenoside Rd	HFD obesity SD mice	i.g. 20 mg/kg for 5 weeks	① Inhibit hepatic gluconeogenesis ② Regulate the composition and function of the intestinal flora	① p-Akt↑, p- Fox01↑, p-GSK3β↑, GLUT2↑, G6pase, PEPCK↓ ② *SMB53*, *rc4-4*, *Ruminococcus*↑	Wang et al. (2023)
	Ginsenoside compound K	HFD T2DM C57BL/6 mice	i.g. 30 mg/kg	Regulate the composition and function of the intestinal flora	NAS, GLP-1↑; *Firmicutes/Bacteroidetes* ratio↓, *Akkermansia*, *Parabacteroides*↑	Huang et al. (2025)
	Ginsenoside Rg5	HFD T2DM C57BL/6 mice	i.g. 50 mg/kg for 2 weeks	Inhibit hepatic gluconeogenesis	PDE4B activity↓, cAMP↓, PEPCK, G6pase↓	Xiao et al. (2017)
	Ginsenoside F4	Male db/db mice	i.g. 45, 90 mg/kg for 6 weeks	Improve insulin resistance	① PTP1B↓, IRE-1/TRAF2/JNK signaling pathway inhibition ② PI3K/AKT signaling pathway activation, GLUT4↑	Zhao et al. (2023)
*Astragalus membranaceus* (Fisch.) Bunge (Fabaceae)	Astragaloside IV	HFD/STZ T2DM KM mice	i.g. 25, 50, 100 mg/kg for 10 weeks	① Improve insulin resistance ② Inhibit the level of oxidative stress ③ Regulate the composition and function of the intestinal flora	① AMPK/SIRT1 signaling pathway and PI3K/AKT signaling pathway activation ② ROS↓ ③ Abundance of SCFAs-producing microorganisms↑	Gong et al. (2021)
	Isoastragaloside I	mPDOs from C57BL/6 mice	10 μM for 14 day	Improve the dysfunction of the pancreatic beta islet cells	① The endocrine progenitor marker Ngn3↑ ② Promotes the differentiation of mPDOs into insulin-secreting	Yu et al. (2022)
*Platycodon grandiflorum* (Jacq.) A. DC. (Campanulaceae)	Platycodin D	HFD/STZ T2DM C57BL/6 J mice	i.g. 2.5, 5.0 mg/kg for 8 weeks	Inhibit hepatic gluconeogenesis	AMPK/ACC/CPT-1 signaling pathway activation, PEPCK1, G6Pase↓	Shen et al. (2023b)
*Dioscorea opposita Thunb.* (Dioscoreaceae)	Dioscin	HFD/STZ T2DM Wistar rats and KK-Ay mice	i.g. 15, 30, 60 mg/kg for 8 weeks	① Improve insulin resistance ② Inhibit hepatic gluconeogenesis	① p-PI3K↑, p-Akt↑, p-Fox01↑ ② miR-125a-5p/STAT3 signaling pathway inhibition, PEPCK↓, G6Pase↓, GSK-3β↑, SREBP-1c↓, FAS↓, ACC↓	Xu et al. (2020)
	The total saponins from *Dioscorea nipponica* Makino	HFD/STZ T2DM Wistar rats	P.O. 50, 100, 200 mg/kg for 12 day	① Improve insulin resistance ② Inhibit hepatic gluconeogenesis ③ Suppress the inflammatory response	① INSR↑, IRS-1↑, p-Akt↑, GLUT-4↑, p-AMPK↑, PPARγ↑, CPT1↑ ②p-GSK3β↓, PEPCK, G6Pase↓ ③ MDA↓, iNOS↓, NO↓, TNF-α↓, IL-6↓, NF-κB↓	Yu et al. (2015)
*Dipsacus asper* Wall. ex DC. [Caprifoliaceae]	Akebia saponin D	HFD/STZ T2DM C57BL/6 J mice	i.g. 50, 100 mg/kg for 4 weeks	Improve insulin resistance	IGF1R/AMPK signaling pathway activation, PI3K/AKT signaling pathway activation, PGC-1α↑	Shu et al. (2024)
*Momordica charantia* L. [Cucurbitaceae]	The ethanol extract of wild bitter gourd	HFD/STZ T2DM Wistar rats	i.g. 150, 300 mg/kg for 4 weeks	① Improve insulin resistance ② Suppress the inflammatory response ③ Inhibit the level of oxidative stress	① AMPK/PI3K signaling pathway activation, p-IRS↑, p-Akt↑, GLUT2↑ ② CRP↓, TNF-α↓, IL-6↓ ③ SOD↑, CAT↑, GSH↑	Sun et al. (2023)
*Polygonatum sibiricum* Redouté (Asparagaceae)	Polygonatum sibiricum saponin	HFD/STZ T2DM ICR male mice	i.g. 1, 1.5, 2 g/kg for 4 weeks	Regulate the composition and function of the intestinal flora	*Firmicutes/Bacteroidetes* ratio↓, the abundance of *Lactobacillus,* and *Intestinimonas*↑	Chai et al. (2021)
*Silene viscidula* Franch. (Caryophyllaceae)	Wacao pentacyclic triterpenoid saponins	HFD T2DM KKAy mice	i.p, 0.5, 1, 2 mg/kg for 30 day	Improve insulin resistance	① PI3K/AKT signaling pathway activation, GLUT4↑ ② Glycolipid metabolism genes: Mogat1↓, Lipc↓, Smpd4↓, Lpcat4↓	Zhang et al. (2021)

Glucose metabolism is predominantly regulated by Akt/FoxO1 and AMPK/Akt signaling pathways, and their dysfunction is mechanistically linked to T2DM pathogenesis. Physiologically, phosphoinositide-3 kinase (PI3K)-dependent phosphorylation of protein kinase B (Akt) Thr308/Ser473 inhibits forkhead box protein O1 (FoxO1) nuclear translocation, disrupting its interaction with peroxisome proliferator-activated receptor gamma coactivator 1α (PGC-1α) and suppressing transcription of gluconeogenic enzymes phosphoenolpyruvate carboxykinase (PEPCK) and glucose-6-phosphatase (G6Pase), thereby reducing hepatic glucose output (Hatting et al., 2017). Ginsenoside Rg1 (Rg1) augments Akt Ser473 phosphorylation, replicating this inhibitory axis (Liu et al., 2017). In addition, ginsenoside Rd (Rd) activates the Akt/GSK3β/GS (glycogen synthase kinase-3β/glycogen synthase) pathway to boost glycogen synthesis, thereby maintaining glucose homeostasis (Wang et al., 2023). Furthermore, dioscin ameliorate glycolipid metabolic dysregulation in T2DM by enhancing glycogen synthesis while suppressing gluconeogenesis and lipogenesis through modulation of the miR-125a-5p/STAT3 pathway, as confirmed by miR-125a-5p and STAT3 overexpression experiments (Xu et al., 2020). Beyond Akt/FoxO1, platycodin D (PD) activates Adenosine 5‘-monophosphate (AMP)-activated protein kinase (AMPK) to phosphorylate sterol regulatory element-binding protein 1 (SREBP-1), acetyl-CoA carboxylase (ACC), and fatty acid synthase (FAS), promoting fatty acid oxidation and suppressing lipogenesis (Hardie et al., 2012), while dioscin further improves lipid metabolism by inhibiting SREBP-1c (Yao et al., 2018). These findings demonstrate how saponins orchestrate hepatic glucose-lipid homeostasis through Akt/FoxO1 and AMPK/Akt signaling, providing a foundation for T2DM therapeutics. However, the majority of these conclusions are derived from hepatocyte cell lines or rodent models. For instance, the regulatory effect of dioscin on the miR-125a-5p/STAT3 axis lacks validation in physiologically relevant human liver models. Moreover, the saponin concentrations employed in many *in vitro* studies (e.g., up to 80 μM) substantially exceed achievable *in vivo* exposure levels, calling into question the physiological relevance of these findings.

### 5.2 Improvement of insulin sensitivity: from cellular signaling to systemic resistance

**FIGURE 4:**
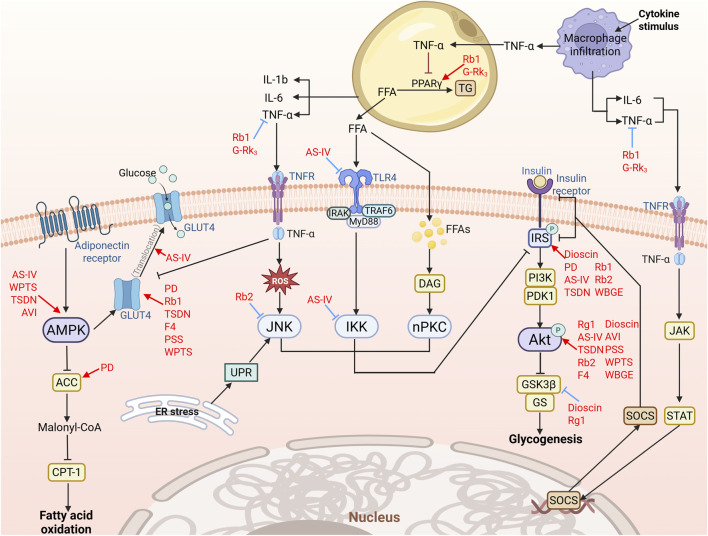
Saponins ameliorate insulin resistance through a multifaceted molecular framework that targets both canonical insulin signaling and complementary metabolic pathways (Created using BioRender). Central to their mechanism is the restoration of insulin sensitivity via direct activation of the IRS-1/PI3K/Akt axis, which enhances glucose uptake through GLUT4 membrane translocation and stimulates glycogen synthesis in insulin-responsive tissues. In parallel, saponins exert potent anti-inflammatory effects by suppressing TLR4/NF-κB and MAPK/JNK signaling cascades, while concurrently activating the AMPK/SIRT1 energy-sensing hub—dual actions that collectively reduce inhibitory serine phosphorylation of IRS-1 and preserve insulin signal fidelity. Further pharmacological interventions include the inhibition of PTP1B, a key negative regulator of insulin signaling, thereby preventing the dephosphorylation of IR and IRS-1 to maintain pathway activity. Additionally, saponins mitigate endoplasmic reticulum (ER) stress by disrupting the IRE-1/TRAF2/JNK axis, which otherwise exacerbates insulin resistance through unresolved protein-folding demands. By coordinately regulating glucose homeostasis (via IRS-1/PI3K/Akt-GLUT4), lipid metabolism (through AMPK/SIRT1), and inflammatory tone (via TLR4/NF-κB/MAPK suppression), saponins emerge as unique polypharmacological agents capable of targeting the metabolic-inflammatory nexus underlying T2DM pathogenesis.

IR is characterized by reduced insulin responsiveness in the liver, adipose issue, and skeletal muscle under euglycaemic hyperinsulinaemia, impairs postprandial glucose uptake and utilization, primarily due to the inhibition of IRS-1/PI3K/Akt signaling pathway through mechanisms involving IRS-1 serine phosphorylation, PI3K attenuation, and Akt dephosphorylation (Ahn, 2025). Herein, we summarize the key pathways by which saponins ameliorate T2DM, primarily through improving insulin sensitivity, in Figure 4 and Table 2. Saponins counteract IR by converging on the energy-sensing hub (SIRT1/AMPK) to enhance insulin sensitivity and glycolipid metabolism, as demonstrated by three saponin—ginsenoside compound K (GCK), Akebia saponin D (ASD), and wild bitter gourd ethanol extract—which activate AMPK/silent information Regulator 2 homolog 1 (SIRT1) and Akt signaling (Huang et al., 2025; Shu et al., 2024; Sun et al., 2023). In skeletal muscle, AS-IV promotes glucose uptake in C2C12 cells via IRS-1/PI3K/Akt activation, glucose transporter type 4 (GLUT4) upregulation, and membrane translocation (Zhu et al., 2016), while *Dioscorea nipponica* Makino (Dioscoreaceae), *Polygonatum sibiricum saponin*, and Wacao pentacyclic triterpenoid saponins likewise enhance glucose utilization through PI3K/Akt-GLUT4 induction (Yu et al., 2015; Chen et al., 2022; Zhang et al., 2021). Moreover, ginsenoside Rg5 (Rg5) restores insulin sensitivity by activating the IRS-1/PI3K/Akt axis, significantly reducing blood glucose (Zhu et al., 2021). These findings indicate that saponins restore metabolic homeostasis through canonical insulin signaling repair (e.g., IRS-1/PI3K/Akt reactivation) and multi-target regulation (e.g., AMPK/SIRT1 activation, GLUT4 modulation), providing a mechanistic basis for further investigation into saponins as insulin resistance-targeting agents, though confirmation in human studies is required. However, critical gaps remain, including tissue-specific effects of saponins, dose optimization for therapeutic windows, and potential synergies with existing antidiabetic drugs (e.g., metformin, GLP-1 agonists), warranting further mechanistic, pharmacokinetic, and translational studies to harness their therapeutic potential in diabetes management fully.

Chronic nutritional excess promotes IR through a complex interplay between inflammatory signaling and lipid metabolism dysregulation. The vicious cycle begins with macrophage infiltration into adipose tissue, triggering excessive release of inflammatory cytokines and free fatty acids (FFAs). These mediators activate IR through distinct but synergistic mechanisms: inflammatory cytokines stimulate MAPK pathways (JNK, IKK, and nPKC) via Toll-like receptor 4 (TLR4) activation, inducing inhibitory serine phosphorylation of IRS proteins, while FFAs exacerbate IR via enhanced lipolysis (Perry et al., 2015; Arneth, 2025). Ginsenoside Rb2 counteracts this cascade by suppressing MAPK signaling, reducing c-Jun N-terminal kinase (JNK)-mediated IRS-1 phosphorylation, and restoring IRS-1/PI3K/Akt insulin signaling (Dai et al., 2018). Early in macrophage activation, PPARγ governs inflammatory cytokine secretion, with both GCK and Rb1 enhancing IRS-1/PI3K expression and insulin sensitivity through PPARγ-targeted activation (Ding et al., 2023; Xu et al., 2022). Notably, the core negative regulator protein tyrosine phosphatase 1B (PTP1B) further exacerbates IR by directly dephosphorylating IR and IRS-1, thereby interrupting PI3K/Akt signaling (Taheripak et al., 2013; Rocha et al., 2021). Intriguingly, dammarane-type triterpenoids from the hydrolyzate of total *Gynostemma pentaphyllum* saponins and ginsenoside F4 inhibit PTP1B while suppressing the IRE-1/TRAF2/JNK axis to relieve skeletal muscle ER stress, thereby reinstating insulin sensitivity (Zhao et al., 2023; Tan et al., 2023). Collectively, saponins counteract IR by simultaneously dampening inflammatory cascades (MAPK/PPARγ) and restoring insulin signaling (PTP1B/IRE-1), highlighting their unique value as multi-target therapeutic agents that simultaneously address metabolic and inflammatory aspects of diabetes pathogenesis.

### 5.3 Suppression of inflammatory responses and attenuation of oxidative stress

**FIGURE 5:**
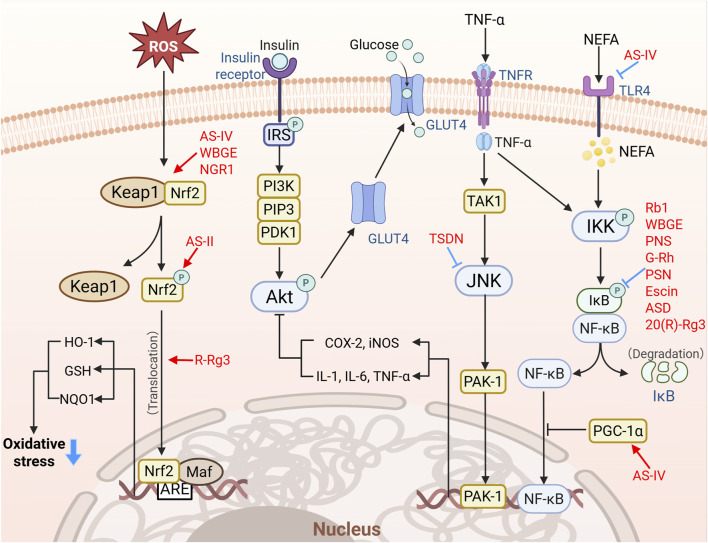
Saponins ameliorate insulin resistance through dual modulation of inflammatory and oxidative stress pathways (Created using BioRender). Saponins target the TLR4/NF-κB axis by inhibiting IKK/IκBα signaling and NF-κB nuclear translocation, thereby suppressing pro-inflammatory cytokines (TNF-α, IL-6). Concurrently, they activate the SIRT1/PGC-1α/Nrf1 mitochondrial pathway and Keap1/Nrf2 axis to upregulate antioxidant enzymes (SOD, CAT) while reducing ROS and MDA levels. Notably, specific saponins (e.g., AS-IV and Rb1) selectively inhibit NOX isoforms and mitigate mitochondrial oxidative damage, restoring metabolic homeostasis in T2DM.

The pathogenesis of obesity-driven T2DM is intrinsically linked to hyperlipidemia, wherein excessive FFAs act as pathogenic mediators by activating TLR4/NF-κB inflammatory signaling in metabolically active tissues such as the liver and skeletal muscle (Arneth, 2025). As summarized in Figure 5 and Table 2, saponins suppress T2DM progression through coordinated inhibition of inflammation and oxidative stress. Mechanistic studies reveal that AS-IV significantly attenuates TLR4 mRNA expression in palmitate-stimulated C2C12 myotubes, inhibits the IκB kinase (IKK)/inhibitor of κB-α (IκBα) signaling cascade, and blocks NF-κB nuclear translocation, thereby reducing cytokine production and restoring insulin sensitivity in T2DM models (Zhu et al., 2016). Furthermore, complementary evidence demonstrates that Rb1 downregulates PPARγ, suppresses NF-κB activation, and mitigates inflammatory cytokine secretion in hypertrophied adipocytes, finally alleviating chronic low-grade inflammation-induced metabolic dysfunction (Ding et al., 2023). Similarly, interventions with the total saponins from *Dioscorea nipponica* Makino and the ethanol extract of wild bitter gourd exert potent anti-inflammatory effects via downregulation of TNF-α and IL-6 expression, and decrease NF-κB and cyclooxygenase-2 (COX-2) protein levels (Yu et al., 2015). Collectively, these findings elucidate the molecular mechanisms through which saponins disrupt the vicious cycle of metabolic inflammation and IR via targeted modulation of the TLR4/NF-κB signaling, underscoring their anti-inflammatory therapeutic potential in diabetes.

Mitochondrial oxidative stress, driven by electron transport chain (ETC) overload under energy surplus, represents a key pathological feature of T2DM targeted by saponins through multiple mechanisms. These bioactive metabolites from saponins exhibit direct ROS-scavenging capacity while upregulating endogenous antioxidants (e.g., SOD, CAT, GSH) and suppressing lipid peroxidation markers such as MDA, as demonstrated by AS-IV, PPD, and WBGE (Sun et al., 2023; Deng et al., 2017; Gong et al., 2021). Saponins also specifically modulate enzymatic ROS sources to suppress oxidative stress, with Rg1 and Rc selectively targeting NADPH oxidase 3 (NOX3) and NOX2 isoforms, respectively (Wang et al., 2021; Fan et al., 2019). At the systemic level, AS-IV coordinates mitochondrial quality control in *db/db* mice through the SIRT1/PGC-1α-mediated pathway, promoting biogenesis through nuclear respiratory factor 1 (Nrf1) and enhancing mitochondrial fusion via mitofusin 2 (Mfn2) (Zhu et al., 2021; Li L. et al., 2024). Notably, astragaloside II (AS-II) and AS-IV concurrently activate the Kelch-like ECH-associated protein 1 (Keap1)/Nrf2 antioxidant axis to induce protective enzymes like heme oxygenase-1 (HO-1) and NAD(P)H quinone dehydrogenase 1 (NQO1) (Shen et al., 2023a; Su et al., 2021). This multilayered antioxidant strategy, including direct neutralization, enzymatic regulation, and transcriptional reprogramming, provides a robust theoretical foundation for targeting oxidative stress in T2DM. However, the anti-inflammatory action of saponins extend beyond single-pathway inhibition. For instance, astragaloside IV (AS-IV) simultaneously suppresses the pro-inflammatory TLR4/NF-κB axis while activating the antioxidant Keap1/Nrf2 pathway. This “dual regulation” likely underpins its high pharmacological effects, yet current research often examines these pathways in isolation, neglecting their complex interplay. Mechanistically, Nrf2 activation can directly antagonize NF-κB signaling by preventing IκBα degradation and inhibiting nuclear translocation of the p65 subunit, thereby establishing an intrinsic feedback loop that links oxidative stress control with inflammatory suppression. Conversely, sustained NF-κB activation may suppress Nrf2 transcriptional activity, creating a pathological vicious cycle that saponins appear to disrupt through concurrent modulation of both pathways (Wardyn et al., 2015). Nevertheless, the precise contribution of Nrf2 activation to the overall anti-inflammatory effect of AS-IV remains unclear and warrants further investigation.

### 5.4 Gut microbiota modulation as a novel therapeutic axis

Emerging evidence underscores gut microbiota dysbiosis as central to T2DM pathogenesis, where dietary imbalances induce structural alterations that disrupt energy homeostasis and promote pro-inflammatory microenvironments (Guo et al., 2022; Ma et al., 2019). Saponins and their microbial metabolites exert multi-dimensional regulation through targeted microbiota remodeling (Table 2). For instance, Rg5 and *Polygonatum sibiricum* saponins (PSS) act as potential prebiotics to reduce *Firmicutes/Bacteroidetes* ratio (F/B ratio) to physiological levels (Chai et al., 2021; Wei et al., 2020); GCK, a gut microbial metabolite of ginsenosides (Rb1, Rb2, Rc, and Rd), enriches *Ruminococcaceae* and elevates the secondary bile acids (e.g., deoxycholic acid), stimulates L-cell proliferation, and enhances the secretion of GLP-1 in db/db mice (Huang et al., 2025; Tian et al., 2022). Additionally, Rb1 enriches beneficial *Parasutterella*, while suppressing pathobionts (*Alistipes* and *Odoribacter*), reduces pro-inflammatory FFAs, and elevates phosphatidylcholine to inhibit TLR4/NF-κB signaling and improve metabolic dysfunction (Zhou R. et al., 2023). These effects are complemented by Rd, AS-IV, and PSS-mediated increases in short-chain fatty acid (SCFA)-producing taxa (*Lactobacillus, Ruminococcus*) and reductions in circulating LPS (Wang et al., 2023; Chen et al., 2022; Gong et al., 2021; Chai et al., 2021), ultimately improving systemic inflammation and hyperglycemia. Key outstanding questions include establishing causal microbiota-metabolism relationships through germ-free models, characterizing saponin pharmacokinetics, addressing interindividual therapeutic variability, and validating clinical translatability beyond rodent systems. Collectively, these findings indicate saponins as precision modulators of the gut-metabolism axis, though bridging mechanistic insights to human applications remains essential for developing next-generation microbiota-targeted T2DM therapy.

### 5.5 Additional pharmacological effects

Pancreatic β-cells, the principal insulin-producing units within pancreatic islets, maintain glucose homeostasis through tightly regulated insulin biosynthesis, storage, and secretion that become critically impaired during T2DM pathogenesis (Rorsman and Ashcroft, 2018). Initially, β-cells compensate for IR via hyperplasia and hypersecretion (Ding et al., 2024), but chronic glucolipotoxicity, oxidative stress, and inflammation lead to functional decline marked by diminished insulin secretion and loss of β-cell mass in T2DM (Marrano et al., 2020). As summarized in Table 2, saponins exhibit therapeutic potential through mechanisms, such as *Aralia* saponins enhance glucose-stimulated insulin secretion via G protein-coupled receptor 40 (GPR40)-mediated Ca^2+^ oscillations and protein kinase C (PKC)-dependent phosphorylation (Cui et al., 2015). Furthermore, Isoastragaloside I promotes β-cell regeneration by upregulating differentiation markers (e.g., Pdx1, MafA) and the progenitor regulator Ngn3 (Yu et al., 2022). Furthermore, saponins from *Aralia taibaiensis* (sAT) and PSS partially reverse structural damage by restoring islet organization and proliferation (Chen et al., 2022; Weng et al., 2014). Nevertheless, the molecular mechanisms underlying saponin-mediated β-cell regeneration and functional recovery remain incompletely elucidated, particularly regarding their effects on β-cell dedifferentiation-redifferentiation balance, mitochondrial functional remodeling, and ER stress regulation. Future studies should integrate multi-disciplinary approaches, such as organoid models and epigenetics, to systematically elucidate saponins’ multi-target effects on β-cell regeneration and functional modulation, thereby advancing therapeutic strategies centered on β-cell functional reconstruction.

In the regulation of postprandial hyperglycemia in T2DM, α-glucosidase, a critical enzyme that hydrolyzes carbohydrates into glucose, represents a major pharmacological target, with clinical inhibitors like acarbose demonstrating pharmacological effects in delaying diabetes progression but often causing dose-dependent gastrointestinal adverse effects (Zhang et al., 2013). This therapeutic limitation is addressed by PSS and Platycodi radix saponin fractions (SK1) potently suppress α-glucosidase to attenuate postprandial hyperglycemia (Lee et al., 2013; Luo et al., 2020). Structure-activity studies have identified promising dammarane triterpenoids from *Cyclocarya paliurus* leaves that exhibit superior inhibitory potency (IC_50_ 257.74∼282.23 μM) compared to the positive control acarbose (IC_50_ 359.36 μM) and effective glucose-lowering action (Li C. et al., 2021). Similarly, sea cucumber-derived triterpenoid glycosides leverage saponin-fatty acid synergism for enhanced pharmacological effects (Puspitasari et al., 2023). Notably, the bark-derived triterpenoid saponins from *Pouteria cambodiana* with C-3 glucuronidation and C-28 oligosaccharide chains inhibit α-glucosidase through dual hydrogen bonding (His280 and Gly309) and hydrophobic interactions at the enzyme’s catalytic pocket, interfering with substrate hydrolysis and delaying intestinal glucose absorption to reduce postprandial hyperglycemia (Sanneur et al., 2023). However, current research remains largely constrained by an overreliance on *in vitro* assays without physiological validation, an incomplete understanding of structure-activity relationships (especially glycosylation patterns and aglycone modifications), and insufficient insight into downstream metabolic consequences. Future studies should employ advanced structural biology techniques (e.g., cryo-EM) to resolve binding dynamics, develop *in situ* intestinal models to evaluate inhibition kinetics, and systematically optimize saponin pharmacophores to translate preclinical findings into clinical applications.

## 6 Saponins in diabetic complications: organ-specific protection

Diabetic vascular complications, the most severe chronic manifestations of T2DM, involve extensive damage to both microvascular and macrovascular systems, constituting the leading cause of disability and mortality in diabetic patients (Tancredi et al., 2015). Microvascular complications include diabetic kidney disease (DKD), diabetic retinopathy (DR), and diabetic cardiomyopathy (DCM), presenting as impaired glomerular filtration, retinal microvascular leakage, and myocardial metabolic abnormalities, respectively. Macrovascular complications involve atherosclerosis-driven pathologies, including coronary artery disease, cerebrovascular stenosis, and peripheral vascular dysfunction, exacerbated by systemic metabolic disturbances (Xue et al., 2023). At the molecular level, persistent hyperglycemia activates NF-κB signaling cascade through the AGEs-RAGE axis, including pro-inflammatory cytokines release (e.g., TNF-α and IL-6) and aberrant NOD-like receptor protein 3 (NLRP3) inflammasome activation, which collectively amplify inflammatory signaling (Westerterp et al., 2018; Tang and Yiu, 2020; Tuleta and Frangogiannis, 2021). These pathological processes disrupt endothelial barrier function, promote vascular permeability, and facilitate lipid deposition, thereby establishing the foundation for progressive vascular complications. Given this complex pathophysiology of T2DM complications, natural saponins have emerged as promising multi-target agents due to their unique capacity to simultaneously modulate redox balance, suppress inflammation, enhance insulin sensitivity, and regulate autophagy (Table 3). Their multifaceted mechanisms of action position them as particularly valuable candidates for preventing and managing both microvascular and macrovascular complications of diabetes.

**TABLE 3:** Mechanism of action of saponins in the treatment of type 2 diabetes complications.

Diabetes complications	Saponin	Study model	Dosing	Pharmaceutical effects	Mechanism	Ref
Diabetic kidney disease	Astragaloside IV	STZ diabetic C57BL/6J rats	i.g. 6 mg/kg for 10 weeks	Improve oxidative stress-induced diabetic kidney injury and podocyte apoptosis	Nrf2-ARE/TFAM signaling pathway activation, HO-1↑, GCLC, GCLM↑, ROS↓	Shen et al. (2023a)
		Glomerular mesangial cells	3, 10, 30 μM for 2 h	Inhibit the proliferation, inflammation, and fibrosis of glomerular mesangial cells	PI3K/Akt and ERK signaling pathway activation, NF-κB signaling pathway inhibition	Su et al. (2023)
		*db/db* experimental mice	i.g. 20 mg/kg for 6 weeks	Reduce renal oxidative stress and improve mitochondrial dysfunction in podocytes	SIRT1/PGC1α/Nrf1 signaling pathway activation	Li L. et al. (2024)
		HFD/STZ SD male rats	i.g. 20, 40, 80 mg/kg for 12 weeks	Regulate lipid metabolism disorders in the kidneys and reduce ectopic lipid deposition	HMOX1/FTH1/TFR1 signaling pathway inhibition, GPX4, ROS↓	Liu et al. (2024b)
		HFD DKD mice	i.g. 10, 20 mg/kg for 8 weeks	Improve renal lipotoxicity and tubular injury	FATP2↓, mtROS↓	Wang J. et al. (2024)
	Ginsenoside Rb1	Streptozotocin-induced DKD mice	i.g. 40 mg/kg for 7 weeks	Alleviate apoptosis of renal podocytes and mitochondrial damage	Aldose reductase activity inhibition, NOX4, ROS↓	He et al. (2021)
	20(R)-Rg3	HFD/STZ T2DM C57BL/6 mice	i.p. 10, 20 mg/kg for 8 weeks	Alleviate oxidative damage to the kidneys by restoring the activity of antioxidant enzymes and inhibiting lipid peroxidation	MAPK signaling pathway and NF-κB signaling pathway inhibition, p-IKKβ↓; ERK, JNK, p38MAPK↓; MDA↓, SOD, HO-1↑	Li Y. et al. (2021)
	Notoginsenoside Fc	C57BL/6J *db/db* mice	i.g. 2.5, 5, 10, 20 mg/kg for 24 h	Improve pyroptosis and mitochondrial dysfunction of glomerular endothelial cells	Drp-1, Fis 1↓, Mfn 2↑, HMGCS2 signaling pathway inhibition	Shen et al. (2024)
	Akebia Saponin D	STZ T2DM C57BL/6 mice	i.g. 50, 100, 150 mg/kg for 8 weeks	Prevent kidney injury, improve renal function and inflammatory response, and inhibit renal tubular cell apoptosis	NRF2/HO-1 signaling pathway activation, NF-κB signaling pathway inhibition, Nrf2, HO-1↑, TNF-α, IL-1β↓, Bax, cleaved caspase-3/9↓, Bcl-2↑	Lu et al. (2020)
Diabetic cardiomyopathy	Platycodin D	HFD/STZ T2DM C57BL/6 mice	P.O. 2.5 mg/kg for 4 weeks	Reduce cardiac damage and enhance the energy metabolism of H9c2 cells	AMPK signaling pathway activation, GLUT4↑, Autophagy-related proteins↑, Bax, cleaved caspase-3↓	Wang W. et al. (2024)
	Astragaloside IV	HFD/STZ T2DM SD rats	i.g. 20, 40, 80 mg/kg for 12 weeks	Improve myocardial injury and myocardial contractile function, and reduce lipid deposition	CD36↓, MDA↓, ROS↓; p53, ACSL4↓; GPX4↑	Li et al. (2023)
​	Ginsenoside Rg1	STZ T2DM C57BL/6 mice and primary cardiac fibroblasts	i.p. 1000 μg/L	Improve cardiac function and reduce myocardial fibrosis	NOTCH signaling pathway activation	Zhen et al. (2023)
	Ginsenoside-Rb1	HFD/STZ T2DM C57BL/6 mice	P.O. 40 mg/kg for 8 weeks	Improve cardiac insufficiency and abnormal Calcium signaling in cardiomyocytes	RyR2, SERCA 2a, and PLN↓inhibit the O-GlcNAc glycosylation of CaMKII and reduce the abnormal phosphorylation of RyR2	Qin et al. (2019)
	Astragaloside IV	HFD/STZ T2DM C57BL/6 mice	i.g. 80 mg/kg for 8 days	Improve myocardial dysfunction and structural damage	TNF-α↓, IL-6, IL-1β↓, TGF-β1↓	Wang et al. (2020)
Diabetic retinopathy	20(R)-ginsenoside Rg3	HFD/STZ T2DM C57BL/6 J mice	I.p. 20 mg/kg for 6 weeks	Reduce retinal barrier damage and thickness changes	Nrf2/HO-1 signaling pathway activation, ER stress↓	Li W et al. (2024)
	Ginsenoside Ro	STZ T2DM C57BL/6 J mice and the Epac1 conditional knockout mouse model	i.g. 90, 225 mg/kg for 6 weeks	Prevent retinal microvascular endothelial damage	ICAM-1, VCAM-1, PVCAM-1↓, IL-1β, TNF-α, IL-6↓, CD31, Bcl-2↓, Epac1/AMPK signaling pathway activation	Liu et al. (2025)
Diabetic neuropathy	Astragaloside IV	HFD GK rats	i.p. 20, 40, 80 mg/kg for 64 h	Reduce peripheral nerve myelin sheath injury and improve nerve function	miR-155↑, PI3K/Akt/mTOR signaling pathway inhibition	Yin et al. (2021)
	Platycodin D	HFD/STZ T2DM C57BL/6 J mice	i.g. 2.5 mg/kg for 4 weeks	Alleviate the oxidative damage to brain tissue and the apoptosis of nerve cells	MDA↓, Nrf2, HO-1↑, ROS↓, Bcl-2↑, PI3K/Akt/GSK3β signaling pathway activation	Lu et al. (2024)
	Ginsenoside Re	HFD/STZ T2DM C57BL/6 J mice	i.g. 5, 10, 20 mg/kg for 4 weeks	Improve brain insulin resistance and cognitive dysfunction	TG, TCHO, LDLC, GOT, GPT↓, HDLC↑, JNK signaling pathway inhibition	Kim et al. (2017)
	Ginsenoside Rg1	HFD/STZ T2DM C57BL/6 J mice	i.g. 1, 5, 10 mg/kg for 8 weeks	Improve memory impairment and neuronal damage	PLC–CN-NFAT1 signaling pathway inhibition, Aβ↓, ROS↓, PSD95, SYN↑	Dong et al. (2023)
Non-alcoholic fatty liver disease	Platycodin D	HFD/STZ T2DM C57BL/6 J mice	i.g. 2.5, 5.10 mg/kg for 8 weeks	Restore abnormal liver function and reduce liver glycogen breakdown	PCK1, G6Pase↓; AMPK/ACC/CPT-1 signaling pathway activation	Shen et al. (2023b)
	Diosgenin	Db/db mice	i.g. 30, 90 mg/kg for 4 weeks	Reduce the intake of fatty acids and lipid accumulation	SIRT6↑, CD36↓, FATP2↓, FABP1↓	Nie et al. (2023)

### 6.1 Diabetic nephropathy (DN): renal protection via saponin-mediated pathways

Diabetic nephropathy (DN), a diabetic microvascular complication, arises from synergistic hyperglycaemic oxidative stress and hypoxia-triggered inflammatory cascades that thicken the glomerular basement membrane, downregulate podocyte proteins, and disrupt the filtration barrier, which leads to progressive proteinuria (Cheng et al., 2020). In rodent models of DN, saponins counteract DN by simultaneously suppressing oxidative stress, inflammation, ferroptosis, and apoptosis, leveraging dual antioxidant mechanisms—ROS generation inhibition and direct radical scavenging. Specifically, AS-IV and AS-II activate the Keap1/Nrf2, promoting Nrf2 nuclear translocation and upregulating downstream antioxidant enzymes (Su et al., 2021; Su et al., 2023). Concurrently, they synergistically enhance cellular oxidative defense through activation of the PI3K/Akt and extracellular regulated protein kinases (ERK) pathways (Shen et al., 2023a), while AS-IV further alleviates oxidative damage in renal tubular epithelial cells by restoring the succinylation of HSD17B10 protein to stabilize the mitochondrial RNase P complex (Wang M. et al., 2024). Given mitochondria’s central role in ROS generation, saponins effectively target mitochondrial dysfunction, as evidenced by AS-IV’s improvement of mitochondrial bioenergetics through activation of the SIRT1/peroxisome proliferator-activated receptor gamma coactivator 1-alpha (PGC1α)/Nrf1 axis to restore ETC complex activity and ATP synthesis (Shen et al., 2023a; Li L. et al., 2024). In addition, gypenoside Fc reduces ROS by modulating mitochondrial dynamics between dynamin-related protein 1 (Drp-1)/mitochondrial fission 1 protein (Fis1) and Mfn2, preserving mitochondrial membrane potential (Shen et al., 2024); meanwhile, Rb1 mitigates oxidative injury in renal tubular podocytes by suppressing the aldose reductase (AR)/NOX4 pathway to reduce ROS accumulation (He et al., 2021). Collectively, saponins act as multi-target DN therapeutics via mitochondrial stabilization and Nrf2 activation. Whereas, critical knowledge gaps remain regarding the relative contributions of different saponin subclasses (e.g., triterpenoid vs. steroidal) to specific antioxidant pathways, optimal intervention timing, mitochondrial–compartment crosstalk, and the pharmacokinetic properties and bioavailability essential for clinical translation.

Chronic low-grade inflammation is a central pathogenic driver of DN, characterized by immune cell infiltration, inflammatory cytokine release, oxidative stress, and fibrosis. Saponins effectively mitigate inflammatory responses to suppress DN development. Mechanistically, AS-IV and notoginsenoside Fc alleviate renal tubular injury and glomerular endothelial cell pyroptosis by suppressing NLRP3 inflammasome activation through fatty acid transport protein 2 (FATP2) modulation (Shen et al., 2024; Wang J. et al., 2024). Additionally, 20(R)-ginsenoside Rg3 synergistically inhibits the MAPK/NF-κB pathway to reduce inflammatory mediators (Li Y. et al., 2021), while dioscin ameliorates inflammation and lipid metabolism by downregulating TNF-α and transforming growth factor-β1 (TGF-β1) (Guo et al., 2018). Beyond inflammation, saponins also mitigate hyperglycemia-induced metabolic disturbances, particularly iron dysregulation and lipid peroxidation that drive ferroptosis in renal tubular cells (Liu et al., 2024a; Liu et al., 2024b). Multiple studies demonstrate that ASD, Rb1, and 20(R)-Rg3 suppress high glucose-induced renal tubular apoptosis by modulating the B-cell lymphoma-2(Bcl-2)/Bcl-2-associated x protein (Bax) ratio and inhibiting caspase-3 activation (He et al., 2021; Li Y. et al., 2021; Lu et al., 2020). Meanwhile, AS-IV alleviates ER stress through PERK-ATF4-CHOP axis regulation to improve renal pathology in diabetic rats (Ju et al., 2019). Despite these advances, future research is warranted to elucidate mechanistic pathway crosstalk and rigorous preclinical evaluation in comorbid models to facilitate clinical translation. Collectively, saponins inhibit DN progression by targeting oxidative damage, inflammation, and fibrosis, providing a theoretical foundation for developing natural product-based multi-pathway therapeutic strategies against DN.

### 6.2 Diabetic cardiomyopathy (DCM): cardioprotective mechanisms

Diabetic cardiomyopathy (DCM), a major diabetes-related cardiovascular complication, is characterized by distinct myocardial structural remodeling and functional impairment independent of coronary artery disease or hypertension. Severe cases progress to heart failure and represent a leading cause of T2DM mortality (Dillmann, 2019). DCM pathogenesis involves a complex interplay of metabolic dysregulation, mitochondrial dysfunction, and aberrant inflammatory activation, collectively driving myocardial damage and functional decline (Miki et al., 2012; Jia et al., 2017; Huynh et al., 2014; Bugger and Abel, 2014). Emerging evidence highlights the therapeutic potential of saponins in targeting these mechanisms through multifaceted interventions. For instance, AS-IV mitigates myocardial lipid accumulation and ferroptosis by downregulating fatty acid transporter CD36 expression (Li et al., 2023), while concurrently suppressing pro-inflammatory cytokine release and TGF-β1-mediated collagen deposition to delay fibrosis (Wang et al., 2020). Also, ophiopogonin D alleviates hyperglycaemic mitochondrial dysfunction—characterized by impaired fusion/fission, defective biogenesis, and dysregulated autophagy—by blocking DRP1 mitochondrial translocation and enhancing fusion protein expression to restore homeostasis (Li W. et al., 2021). In contrast, PD enhances autophagy through AMPK/mTOR pathway activation while suppressing apoptosis via AMPK-dependent modulation of caspase cascades and Bcl-2/Bax balance (Wang W. et al., 2024). Beyond metabolic and mitochondrial repair, saponins exhibit immunomodulatory properties. Specifically, Rg1 promotes M2 macrophage polarization via mesenchymal stem cell (MSC)-derived exosome circNOTCH1 delivery to attenuate the pro-inflammatory microenvironment in DCM (Zhen et al., 2023). Meanwhile, Rb1 restores sarcoplasmic reticulum calcium homeostasis by modulating Ryanodine Receptor 2 (RyR2) and the sarcoplasmic/endoplasmic reticulum Ca^2+^ ATPase 2a (SERCA2a) activity to mitigate calcium dysregulation (Qin et al., 2019). These findings demonstrate that saponins intervene in key pathological nodes of DCM through integrated modulation of metabolic remodeling, mitochondrial repair, immunomodulation, and calcium homeostasis, offering insights for developing anti-heart failure therapies with dual metabolic regulation and cardioprotective functions. However, clinical translation of saponins for DCM therapy necessitates pharmacokinetic optimization, long-term safety profiles, and potential synergistic interactions with existing therapies.

### 6.3 Neuroprotection: combating neuropathy and cognitive decline

Diabetic peripheral neuropathy (DPN) is characterized by progressive peripheral nerve damage and central cognitive impairment resulting from hyperglycemia-induced oxidative stress, chronic inflammation, and impaired synaptic function (Callaghan et al., 2012). In preclinical studies using rodent models of type 2 diabetes and cultured neuronal or Schwann cell lines, saponins exhibit unique neuroprotective properties by addressing the above mechanisms synergistically. For example, AS-IV mitigates hyperglycemia-induced peripheral nerve demyelination and central cognitive dysfunction by activating the Nrf2/Keap1 pathway to reduce oxidative damage while suppressing pro-inflammatory cytokine release (Zhang et al., 2022). Furthermore, AS-IV enhances Schwann cell autophagy via miR-155-mediated PI3K/Akt/mTOR inhibition, restoring neural conduction (Yin et al., 2021). Neuroinflammation, a key driver of DPN progression, is targeted by timosaponin through Aβ reduction and TNF-α suppression (Liu et al., 2012), while escin blocks NF-κB signaling in peripheral nerves (Suryavanshi and Kulkarni, 2020). CK and dioscin suppress NLRP3 inflammasome activation via distinct mechanisms—ER stress alleviation and P2X7R downregulation, thereby reducing hippocampal microglial activation and neuronal injury (Li et al., 2020; Lu et al., 2021). Ginsenoside Re corrects JNK-mediated insulin signaling abnormalities in neurons (Kim et al., 2017), whereas Rg1 normalizes calcium homeostasis via PLCε-Ca^2+^-CN-NFAT1 inhibition and enhances synaptic plasticity through synapsin/PSD-95 upregulation (Dong et al., 2023). PD further protects neurons from hyperglycemia-induced apoptosis by maintaining mitochondrial membrane potential in HT22 cells and activating the PI3K/Akt/GSK3β signaling (Lu et al., 2024). Preclinical evidence suggests that saponins may serve as multi-target agents for DPN by concurrently ameliorating oxidative stress, neuroinflammation, and synaptic dysfunction, warranting further translational investigation. Future research should focus on evaluating saponin blood-brain barrier permeability and long-term safety to facilitate the translation of multi-target interventions into precision therapies.

### 6.4 Ocular complications: retinopathy and cataract prevention

Diabetic retinopathy (DR) is driven by sustained hyperglycemic memory, which perpetuates oxidative stress, low-grade inflammation, and mitochondrial dysfunction, ultimately disrupting retinal vascular integrity and neuroglial architecture. Oxidative stress in DR triggers mitochondrial defects, apoptosis, inflammation, and lipid peroxidation. Mechanistically, ginsenoside Rg3 activates the Nrf2/Keap1 pathway to upregulate HO-1, which alleviates oxidative and ER stress-induced endothelial injury (Li W. et al., 2024); meanwhile, Rd suppresses NOX2 activation through AMPK/AR signaling, reducing ROS and retinal vascular leakage (Tang et al., 2022). Inflammation, a central pathological mechanism in DR, is attenuated by *Panax notoginseng* saponins through NF-κB inhibition to restore blood-retinal barrier function and mitigate microvascular dysfunction (Wang Y. et al., 2024). Notably, Rg1 further ameliorates abnormal vascular permeability and delays DR progression by suppressing NLRP3 inflammasome activation, downregulating p-NF-κB and VEGF expression, and modulating TRPC6/NFAT2 signaling (Li B. et al., 2022). Mitochondrial dysfunction, a critical contributor to DR pathogenesis, is counteracted by ginsenoside Ro through Epac1/AMPK-mediated mitophagy restoration (Liu et al., 2025), while notoginsenoside R1 enhances the PINK1/Parkin-dependent mitophagy, reducing p62/SQSTM1 accumulation and stabilizing mitochondrial membrane potential (Zhou et al., 2019). In summary, these findings confer multi-target protection against DR through synergistic antioxidant, anti-inflammatory, and autophagic mechanisms. Future research should prioritize evaluating ocular tissue targeting and long-term safety profiles to facilitate the clinical translation of these interventions.

T2DM is strongly linked to non-alcoholic fatty liver disease (NAFLD), with its advanced form, non-alcoholic steatohepatitis (NASH), posing risks of hepatocellular carcinoma and decompensation (Kim and Lee, 2020; Sumida et al., 2020). IR-induced lipolysis increases circulating FFAs, driving hepatic steatosis, oxidative stress, and inflammation. Excessive ROS and pro-inflammatory cytokines accelerate NASH progression toward fibrosis and hepatocellular carcinoma. Pharmacologically, PD ameliorates hepatic steatosis in T2DM by activating the AMPK/ACC/carnitine palmitoyltransferase 1 (CPT-1) pathway to suppress lipogenesis and enhance β-oxidation (Shen et al., 2023b). Similarly, diosgenin upregulates SIRT6, downregulating fatty acid transporters (such as CD36, FATP2, and FABP1) to improve lipid metabolism and reduce hepatic lipid uptake, thereby lowering serum triglycerides, cholesterol, and liver enzyme levels (Nie et al., 2023). Despite these promising findings, current research remains fragmented, focusing on single pathways rather than the integrative mechanisms of saponins across glucolipid metabolism, mitochondrial function, and immune regulation. Future studies should employ multi-stage animal models to systematically evaluate saponins’ synergistic effects, with emphasis on glucolipid metabolism regulation, mitochondrial functional restoration, and immune microenvironment modulation to advance targeted, multi-model therapies for diabetic liver disease.

## 7 Conclusion

Saponins, as key bioactive metabolites from medicinal plants, exhibit multi-target pharmacological activities in preclinical models of T2DM and its complications (diabetic neuropathy, diabetic kidney disease, diabetic retinopathy, and diabetic cardiomyopathy) by simultaneously addressing core pathological mechanisms, including glucolipid metabolic dysregulation, IR, oxidative stress, and chronic inflammation. These metabolites exert systemic effects through: (1) molecular-level modulation of critical pathways (Keap1/Nrf2, AMPK/PI3K/Akt, and NF-κB signaling); (2) functional restoration of mitochondrial dynamics; and (3) gut microbiota remodeling via the gut-organ axis, which enhances SCFA production while attenuating LPS-induced inflammation. Their organ-protective pharmacological effects are particularly evident in diabetic complications, preserving podocyte integrity in nephropathy, optimizing myocardial bioenergetics, restoring neural plasticity, and inhibiting pathological angiogenesis in retinopathy, highlighting their advantages over single-target hypoglycemic agents. Despite promising preclinical data, key barriers hinder saponin clinical translation: (1) mechanistic gaps—synergistic saponin interactions (e.g., ginsenoside and astragaloside) and stage-specific effects remain uncharacterized; (2) PK/PD limitations—low bioavailability requires glycosyl modification and nanoparticle/exosome delivery systems; (3) clinical deficits—lack of phase-adaptive trials assessing long-term safety and drug combinations. AI-guided multi-omics for structure optimization, targeted delivery platforms, and stratified clinical trials to advance saponins as next-generation multi-target therapies.

## Glossary

ACC: Acetyl-CoA carboxylase
AC: Adenylate cyclase
Akt: Protein kinase B
AMPK: AMP-activated protein kinase
ARE: Antioxidant response element
ATP: Adenosine triphosphate
Bax: BCL2-associated X protein
Bcl-2: B-cell lymphoma 2
cAMP: Cyclic adenosine monophosphate
CAT: Catalase
CD36: Cluster of differentiation 36
CoA: Coenzyme A
COX-2: Cyclooxygenase-2
CREB: cAMP response element-binding protein
CPT-1: Carnitine palmitoyltransferase 1
DAG: Diacylglycerol
DKD: Diabetic kidney disease
ELSD: Evaporative light scattering detection
ER: Endoplasmic reticulum
ERK: Extracellular signal-regulated kinase
FAS: Fatty acid synthase
FFA: Free fatty acid
FoxO1: Forkhead box protein O1
G6Pase: Glucose-6-phosphatase
GAPDH: Glyceraldehyde-3-phosphate dehydrogenase
GCLM: Glutamate-cysteine ligase modifier subunit
GLP-1: Glucagon-like peptide-1
GLUT2: Glucose transporter 2
GLUT4: Glucose transporter type 4
GSK3β: Glycogen synthase kinase 3 beta
GTP: Guanosine triphosphate
HFD: High-fat diet
HIF-1α: Hypoxia-inducible factor 1-alpha
HO-1: Heme oxygenase-1
HPLC: High-performance liquid chromatography
HPTLC: High-performance thin-layer chromatography
HPLC-ELSD: High-performance liquid chromatography-evaporative light scattering detection
i.g.: Intragastric administration
i.p.: Intraperitoneal injection
IKK: IκB kinase
IL-1β: Interleukin-1 beta
IL-6: Interleukin-6
iNOS: Inducible nitric oxide synthase
IRAK: Interleukin-1 receptor-associated kinase
IR: Insulin resistance
IRS: Insulin receptor substrate
JNK: C-Jun N-terminal kinase
Keap1: Kelch-like ECH-associated protein 1
LKB1: Liver kinase B1
LPS: Lipopolysaccharide
MAPK: Mitogen-activated protein kinase
MCP-1: Monocyte chemoattractant protein-1
MPC1: Mitochondrial pyruvate carrier 1
mTOR: Mechanistic target of rapamycin
MyD88: Myeloid differentiation primary response 88
NADPH: Nicotinamide adenine dinucleotide phosphate
NF-κB: Nuclear factor kappa-light-chain-enhancer of activated B cells
NO: Nitric oxide
nPKC: Novel protein kinase C
NQO1: NAD(P)H quinone dehydrogenase 1
Nrf2: Nuclear factor erythroid 2-related factor 2
PAK-1: P21-activated kinase 1
PC: Pyruvate carboxylase
PDH: Pyruvate dehydrogenase
PDK1: Phosphoinositide-dependent kinase-1
PEPCK: Phosphoenolpyruvate carboxykinase
PGC-1α: Peroxisome proliferator-activated receptor gamma coactivator 1-alpha
PI3K: Phosphatidylinositol 3-kinase
PIP3: phosphatidylinositol (3,4,5)-trisphosphate
PKA: Protein kinase A
P.O.: Per os (oral administration)
PPARγ: Peroxisome proliferator-activated receptor gamma
PTP1B: Protein tyrosine phosphatase 1B
ROS: Reactive oxygen species
SCFAs: Short-chain fatty acids
SIRT1: Sirtuin 1
SOCS: Suppressor of cytokine signaling
SOD: Superoxide dismutase
SREBP1c: Sterol regulatory element-binding protein 1c
STAT: Signal transducer and activator of transcription
STZ: Streptozotocin
T2DM: Type 2 diabetes mellitus
TAK1: Transforming growth factor-beta-activated kinase 1
TCA cycle: Tricarboxylic acid cycle
TGF-β1: Transforming growth factor-beta 1
TLR4: Toll-like receptor 4
TNF-α: Tumor necrosis factor-alpha
TNFR: Tumor necrosis factor receptor
TRAF6: TNF receptor-associated factor 6
UPLC-MS/MS: Ultra-performance liquid chromatography-tandem mass spectrometry
UPR: Unfolded protein response
